# A Mitochondria‐Related Signature in Diffuse Large B‐Cell Lymphoma: Prognosis, Immune and Therapeutic Features

**DOI:** 10.1002/cam4.70602

**Published:** 2025-01-15

**Authors:** Zhen‐Zhong Zhou, Jia‐Chen Lu, Song‐Bin Guo, Xiao‐Peng Tian, Hai‐Long Li, Hui Zhou, Wei‐Juan Huang

**Affiliations:** ^1^ Department of Medical Oncology Sun Yat‐sen University Cancer Center Guangzhou China; ^2^ State Key Laboratory of Oncology in South China, Guangdong Provincial Clinical Research Center for Cancer Sun Yat‐sen University Cancer Center Guangzhou China; ^3^ Department of Respiratory Oncology, the First Affiliated Hospital of USTC, Division of Life Sciences and Medicine University of Science and Technology of China Hefei Anhui China; ^4^ Department of Pharmacology, College of Pharmacy Jinan University Guangzhou China

**Keywords:** diffuse large B‐cell lymphoma, drug sensitivity, gene set enrichment analysis, immune environment, mitochondria‐related genes, prognostic model

## Abstract

**Background:**

Distinctive heterogeneity characterizes diffuse large B‐cell lymphoma (DLBCL), one of the most frequent types of non‐Hodgkin's lymphoma. Mitochondria have been demonstrated to be closely involved in tumorigenesis and progression, particularly in DLBCL.

**Objective:**

The purposes of this study were to identify the prognostic mitochondria‐related genes (MRGs) in DLBCL, and to develop a risk model based on MRGs and machine learning algorithms.

**Methods:**

Transcriptome profiles and clinical information were obtained from the Gene Expression Omnibus (GEO) database. The risk model was defined using Least Absolute Shrinkage and Selection Operator (Lasso) regression algorithm, and its prognostic value was further examined in independent datasets. Patients were stratified into two clusters based on the risk scores, additionally a nomogram was generated based on the risk score and clinical characteristics. Gene pathway level, microenvironment, expression of targeted therapy‐associated genes, response to immunotherapy, drug sensitivity, and somatic mutation status were compared between clusters.

**Results:**

Eighteen prognostic MRGs (DNM1L, PUSL1, CHCHD4, COX7A1, CPT1A, CYP27A1, POLDIP2, PCK2, MRPL2, PDK3, PDK4, MARC2, ACSM3, COA7, THNSL1, ATAD3B, C15orf48, TOMM70A) were identified to construct the risk model. Remarkable discrepancies were observed between groups. The high‐risk group had shorter overall survival, less immune infiltration, lower CD20 and higher PD‐L1 expression than the low‐risk group. Distinct immune microenvironment, responses to immunotherapy and predictive drug IC50 values were found between groups.

**Conclusions:**

We established a novel prognostic mitochondria‐related signature by machine learning algorithm, which also demonstrated outstanding predictive value in tumor microenvironment and responses to therapies.

## Graphical abstract

This study developed a mitochondria‐related genes prognostic model using machine learning algorithm. The prognostic model exhibits promising prognostic values and has the ability to provide evidence for the selection of appropriate targeted therapy and immunotherapy.

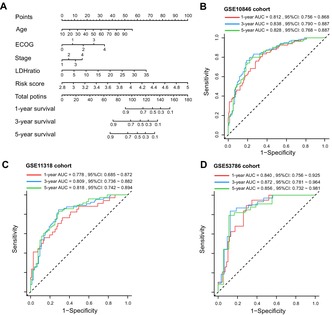

## 1 Introduction

Diffuse large B‐cell lymphoma (DLBCL) is a frequent form of NHL, comprising about 30%–35% of malignant lymphomas [1]. The R‐CHOP regimen is one of the most widely used clinical treatments for this type of lymphoma, which has led to remission or even cures in many patients. However, given to the substantial heterogeneity observed in DLBCL, there remains a significant disparity in post‐treatment outcomes. This indicates that even patients with identical clinicopathological characteristics, such as stages, LDH levels and cell of origin (COO) types, can exhibit markedly disparate responses to the same treatment [2].

Mitochondrion is an organelle involved in a variety of cellular activities [3], primarily producing adenosine triphosphate (ATP). Numerous studies have demonstrated that mitochondrion plays a crucial role in cancer initiation, progression, immune microenvironment and drug resistance [4, 5]. For example, by producing abnormal amounts of reactive oxygen species (ROS) and provoking other dysregulations, mitochondria promote cell proliferation and contribute to the enhancement of anti‐apoptotic mechanisms and the counteraction of other cell death pathways [6, 7, 8]. Previous studies have indicated a tight correlation between DLBCL and mitochondrial dysfunction, suggesting that mitochondria could be a new therapeutic target for DLBCL [9, 10]. For instance, SIRT3, one of the most critical mitochondrial lysine deacetylases, is essential for DLBCL cell's survival [11, 12]. SIRT3 depletion disrupts acetyl‐CoA pools, triggering multiple forms of cell death in DLBCL cells. Furthermore, the elevated expression of specific mitochondrial genes is found to be closely correlated with a unique subtype of DLBCL, OxPhos‐DLBCL, which shows higher tolerance to reactive oxygen species (ROS) toxicity than non‐OxPhos DLBCLs and demonstrates resistance to BCL6 inhibitors [13, 14]. Also, the activation of PDP1 (pyruvate dehydrogenase phosphatase), a pivotal mitochondrial enzyme, has recently been shown to provoke resistance to ibrutinib [15].

Moreover, immunotherapy is receiving increasing attention in DLBCL management, but it is still unclear which set of patients may benefit from it [16, 17, 18]. Exploring mitochondria‐related prognostic markers and targeting them may help improve the efficacy of treatments and offer a more comprehensive understanding to the DLBCL biology.

In this study, an 18 mitochondria‐related genes (MRGs) model was established to access the outcomes of DLBCL patients, as well as a nomogram encompassing clinical characteristics and risk scores to predict overall survival (OS) for DLBCL patients. We next conducted subsequent analyses to explore the discrepancies in the immune microenvironment, biological pathways, drug sensitivities, and mutational profiles among patient subsets. Finally, our findings suggest that the MRGs hallmark is effective at predicting prognosis in DLBCL, providing new insights into its diagnosis and therapy.

## 2 Methods

### 2.1 Data Acquisition

Expression array files encompassing the training cohort (GSE10846) and validation cohorts (GSE11318 and GSE53786) were collected from the Gene Expression Omnibus database (GEO). Associated clinical information was also obtained, encompassing overall survival data, clinical staging, sex, age, LDH ratio, and other relevant factors. In GSE10846, 412 patients met the eligibility criteria for this study (overall survival time > 0), and their transcriptome data were used for further analysis. The GSE11318 dataset included 199 qualified patients, and the GSE53786 dataset comprised 119 qualified patients.

MitoCarta3.0 database (http://www.broadinstitute.org/mitocarta) contains 1136 genes involved in sub‐mitochondrial localization and mitochondrial pathway annotations [19], from which we extracted the MRGs. The clinical information for the training and validation sets is detailed in the Data S1. The training cohort was utilized to screen for the potential prognostic MRGs and generate the risk predictive model, while validation sets were used to verify the capacity for prediction of the model.

### 2.2 Screening the Prognosis‐Related Genes and Enrichment Analysis

Lasso‐Cox regression analysis and cross‐validation (10‐fold) were applied to the training set, candidate MRGs were retrieved based on the minimum error lambda value plus one standard error (lambda 1‐s.e) using the R package “glmnet”. Subsequently, we performed enrichment analyses such as Gene Ontology (GO) and KEGG on the selected genes using the R package “clusterProfiler”. Using the Benjamini‐Hochberg correction (or FDR correction), the original *p* values were adjusted to control for false discovery rates across multiple hypothesis tests to ensure statistical significance. Finally, pathways with adjusted *p* values less than 0.05 were selected and visualized using the R package “ggplot2”. To elucidate the intrinsic mechanisms underlying the MRGs even further, protein–protein interactions (PPI) analysis was performed using the STRING database. Only networks with interaction scores ≥ 0.7 were included for high‐quality results. Clusters were identified using the k‐means algorithm integrated within the database.

### 2.3 Risk Scoring Model Construction

The risk model was constructed on the candidate MRGs selected by the Lasso‐Cox analysis. Ultimately, 18 MRGs were employed to generate the risk scoring model according to a specific mathematical formula and coefficients obtained from the Lasso algorithm. Patients were assigned into low‐ and high‐risk clusters based on the risk score calculated by the prognostic model, and the optimal cut‐off value was determined using the X‐tile 3.6.1 software.

### 2.4 Evaluation of the Value of the Risk Scoring Model

Kaplan–Meier (K‐M) survival analysis and the 1‐, 3‐ and 5‐year operating characteristic (ROC) curves were applied to access the predictive performance of the risk model, using the R packages “survival” and “timeROC”. In order to further access the general applicability, we chose GSE11318 and GSE53786 datasets as validation cohorts. The distribution of patients in the subsets across the three datasets was visualized using the R package “ggplot2”.

### 2.5 Developing of Nomogram Signature

Independent and significant characteristics were identified between the risk score and other conventional clinical features by applying univariate and multivariate Cox analyses sequentially. Based on the major characteristics identified by multivariate Cox analysis, a nomogram was then generated to predict OS at 1, 3 and 5 years. We also assessed the accuracy and general applicability of the nomogram with the validation sets above.

### 2.6 Differential Expression and GSEA Analyses

Gene expression array data was logarithmic normalized first, then differential gene (DEG) analysis was conducted, using the R package “limma”. DEGs with adjusted *p* values less than 0.05 and log‐fold changes exceeding 1 were considered to be statistically significant and thus employed for GSEA enrichment.

### 2.7 Immunological Differences Analysis

To test if the model could predict the feature of microenvironment, we explored the composition of the immune environment using single‐sample gene set enrichment analysis (ssGSEA). Additionally, CIBERSORT and TIDE analyses were employed to established the differences in the macrophage composition and responses to immunotherapy between groups. The ssGSEA algorithm, a subtype of gene set enrichment analysis, was designed to estimate genome enrichment in individual samples rather than in groups of samples. The gene set we applied was obtained from a formal study [20], which contains transcriptomic signatures for tumor‐infiltrating immune cells (TICs). The gene set is publicly accessible at http://cis.hku.hk/TISIDB. The enrichment scores of TICs among patient subgroups were computed and visualized. We also investigated the connection among the expression of 18 MRGs, the risk score, and the enrichment scores of TICs. Given the intricate roles played by different types of macrophages in DLBCL, we investigated the macrophage component using the CIBERSORT algorithm. The tumor immune dysfunction and exclusion (TIDE) scores were established to access the effectiveness of immune checkpoint inhibitors (ICIs) in both groups. Furthermore, we evaluated the differential expression levels of immunotherapy and targeted‐therapy associated molecules across subsets using wilcoxon test.

### 2.8 Drug Sensitivity Analysis

Differential IC50 values of 198 compounds among subgroups of patients were retrieved with the R package “oncoPredict”. Statistically significant disparities in IC50 values among groups were identified with the *p* value threshold of less than 1e‐8. The associations between the IC50 values of the compounds that were found to be significant and the risk score were also investigated.

### 2.9 Mutational Analysis

Somatic mutation data were downloaded from The Cancer Genome Atlas (TCGA) database (https://portal.gdc.cancer.gov/) in mutation annotation format (MAF) to investigate the mutational differences across the patients. MAF files were analyzed through the R package “maftools”.

### 2.10 Statistical Analysis

This study employed R software (v4.0.0) for the bioinformatics and statistical analyses. The wilcoxon test was used to detect statistically significant differences, specifically when comparing the expression levels of targeted therapy and immunotherapy associated molecules across subgroups. The likelihood of survival was measured by the log‐rank test. Correlations, such as the relationships among enrichment scores of TICs, MRGs expression and risk scores, were accessed by Spearman correlation analysis.

## 3 Results

### 3.1 Identifying the Prognostic Genes

**FIGURE 1:**
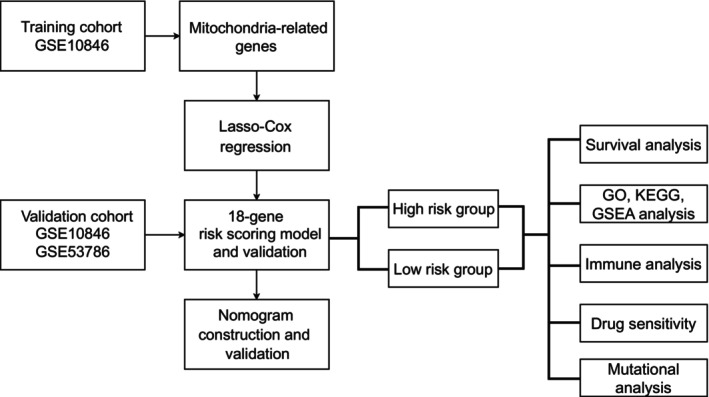
Study flowchart. This flowchart outlines the study design. Mitochondria‐related genes were extracted from the Human MitoCarta 3.0 database. An 18 MRGs classifier was constructed in the training set using Lasso‐Cox regression. Validation analyses were conducted with external independent datasets (GSE11318, GSE53786). A risk scoring signature was used to classify patients into high‐ and low‐risk groups. Further analyses included immune infiltration analyses, survival analyses, enrichment analyses, drug sensitivity analyses, and mutation analyses.

**FIGURE 2:**
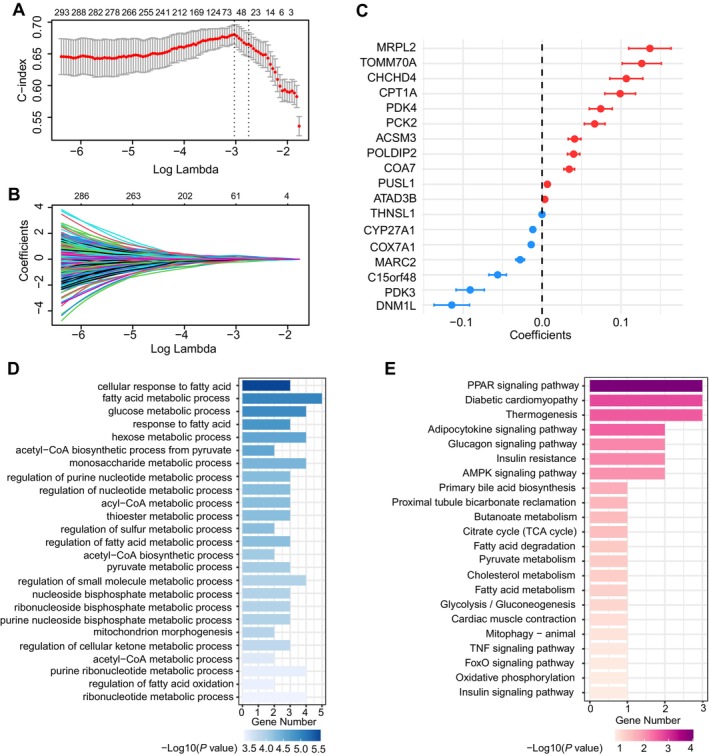
Construction of the mitochondria‐related 18‐gene prognostic model. (A) C‐index of the Lasso‐Cox regression model with two dotted vertical lines indicating the optimal values determined by minimum and 1‐s.e. criteria. (B) Distribution of coefficients from the Lasso‐Cox regression. (C) The distribution of the coefficients of the 18 genes selected by the Lasso‐Cox regression. (D) Bar plot illustrating results of GO enrichment analyses for genes selected by the Lasso‐Cox regression. (E) Bar plot illustrating the results of KEGG enrichment analyses for genes selected by the Lasso‐Cox regression.

The overall process of our study is depicted in Figure 1. The expression profiles of the MRGs were isolated on the basis of the training cohort. In order to ascertain the key prognostic genes, Lasso‐Cox regression algorithm was applied to the training cohort. Consequently, 18 genes (DNM1L, PUSL1, CHCHD4, COX7A1, CPT1A, CYP27A1, POLDIP2, PCK2, MRPL2, PDK3, PDK4, MARC2, ACSM3, COA7, THNSL1, ATAD3B, C15orf48, TOMM70A) were identified as prognostic MRGs. In Figure 2A, the dashed line on the right corresponds to a lambda 1‐s.e. of the Lasso model, which yields a strong predictive effect. In Figure 2B each line represents an MRG and the endpoints indicate the values of the matching coefficients. Figure 2C provides a detailed illustration of the coefficients' distribution for the 18 MRGs. Subsequently, GO and KEGG analyses were employed on the 18 MRGs. As displayed in Figure 2D,E, terms and pathways were inferred to be significantly correlated if the associated *p* values were less than 0.05. For GO terms enrichment, 18 MRGs were specifically enriched in biological processes like fatty acid associated metabolic processes, acetyl−CoA associated metabolic processes, nucleotide associated metabolic processes and purine ribonucleotide metabolic processes. While for KEGG enrichment, 18 MRGs were mainly enriched in pathways including PPAR signaling pathway, diabetic cardiomyopathy, thermogenesis, etc. The PPI networks revealed complex interactions between the 18 MRGs and proteins predominantly involved in mitochondrial respiratory chain complex IV, glycolytic process, citrate cycle (TCA cycle), regulation of pyruvate dehydrogenase (PDH) complex, pseudouridine synthesis, RNA polymerases D, superoxide anion generation, as illustrated in Figure S1.

### 3.2 Constructing the Prognostic Model for 18 Mitochondria‐Related Genes

The processes of constructing and validating the risk scoring model are described in this part. The 18 MRGs selected by the Lasso algorithm were assembled into a risk model, including DNM1L, PUSL1, CHCHD4, COX7A1, CPT1A, CYP27A1, POLDIP2, PCK2, MRPL2, PDK3, PDK4, MARC2, ACSM3, COA7, THNSL1, ATAD3B, C15orf48 and TOMM70A. Finally, the scoring formula was generated according to the expression profiles of the 18 MRGs and their corresponding coefficients. The risk score was calculated based on the formula below: risk score = (−0.1141 × Expression level of DNM1L) + (0.0067 × Expression level of PUSL1) + (0.1067 × Expression level of CHCHD4) + (−0.0138 × Expression level of COX7A1) + (0.0989 × Expression level of CPT1A) + (−0.0117 × Expression level of CYP27A1) + (0.0400 × Expression level of POLDIP2) + (0.0666 × Expression level of PCK2) + (0.1366 × Expression level of MRPL2) + (−0.0911 × Expression level of PDK3) + (0.0743 × Expression level of PDK4) + (−0.0278 × Expression level of MARC2) + (0.0412 × Expression level of ACSM3) + (0.0344 × Expression level of COA7) + (−0.0001 × Expression level of THNSL1) + (0.0034 × Expression level of ATAD3B) + (−0.0562 × Expression level of C15orf48) + (0.1263 × Expression level of TOMM70A).

### 3.3 Evaluation of the Prognostic Model

**FIGURE 3:**
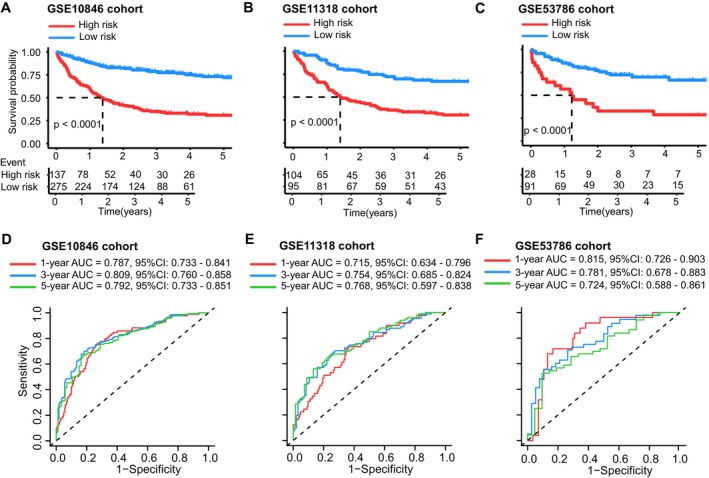
Kaplan–Meier survival curves and time‐dependent ROC curves of the 18‐gene classifier in the training, and external independent validating datasets. (A) Kaplan–Meier curves of overall survival (OS) for high‐risk and low‐risk groups in the training cohort, including distribution between the two groups with the mean value (vertical dashed line) of the risk score, and distribution of survival status in high‐ and low‐risk groups in the training cohort (GSE10846). (B) Kaplan–Meier curves of OS for high‐risk and low‐risk groups in the GSE11318 cohort, including distribution between the two groups with the mean value (vertical dashed line) of the risk score in GSE11318, and distribution of survival status in high‐ and low‐risk groups in GSE11318. (C) Kaplan–Meier curves of OS for high‐ and low‐risk groups in GSE53786, including distribution between the two groups with the mean value (vertical dashed line) of the risk score in GSE53786, and distribution of survival status in high‐ and low‐risk groups in GSE53786. (D) ROC curves for the training cohort. (E) ROC curves for GSE11318 cohort. (F) ROC curves for GSE53786 cohort.

In this section, KM analyses and ROC curves were applied to estimate the prognostic model. Patients were allocated into low‐ and high‐risk groups based on their risk scores, the optimal cut‐off value was 3.9. In Figure 3A–C, the KM curves of the training cohort and validation cohorts are illustrated. Significant differences (*p* < 0.0001) in overall survival across subsets of patients were observed according to our findings. Figure 3D–F illustrate the ROC curves for 1, 3, and 5 years across three cohorts. The area under curves (AUC) values at 1, 3, and 5 years were 0.787 (95% CI: 0.733–0.841), 0.809 (95% CI: 0.760–0.858), and 0.792 (95% CI: 0.733–0.851), respectively in the training cohort. In the GSE11318 cohort, the AUC values for these time points were 0.715 (95% CI: 0.634–0.796), 0.754 (95% CI: 0.685–0.824), and 0.768 (95% CI: 0.597–0.838), respectively. Similarly, the GSE53786 cohort showed AUC values of 0.815 (95% CI: 0.726–0.903), 0.781 (95% CI: 0.678–0.883), and 0.724 (95% CI: 0.588–0.861) for 1, 3, and 5 years, respectively. Based on the above data, we can infer that the 18 MRGs risk model is highly generalizable and has the potential to be used to provide accurate prognoses in DLBCL. The distributional characteristics of patients' risk scores and OS are summarized in Figure S2.

### 3.4 Construction and Validation of Nomogram Model

**FIGURE 4:**
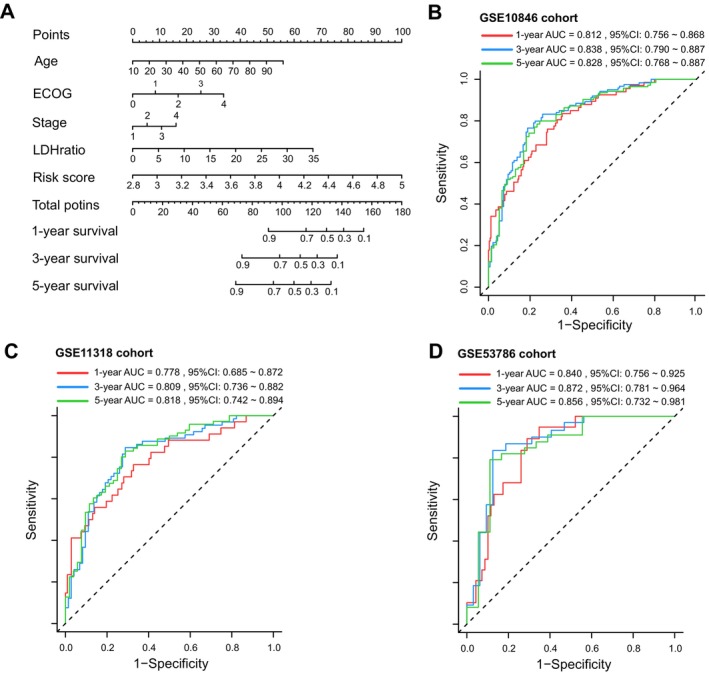
Construction of the nomogram model and time‐dependent ROC curves validation. (A) Nomogram to predict the 1‐, 3‐ and 5‐year of survival in DLBCL. (B) ROC curves for the training cohort. (C) ROC curves for GSE11318 cohort. (D) ROC curves for GSE53786 cohort.

The nomogram was developed from conventional clinical factors and the risk score. The clinical information was extracted from three datasets, detailed information is available in Data S1. First, we applied univariate Cox analysis to the risk score and conventional clinical attributes. As a result, ECOG performance, LDH ratio, age, stage, extra nodal site numbers and the risk score were statistically significant (*p* < 0.05). Subsequently, multivariate Cox analysis was conducted on the former result. Finally, age, ECOG performance, stage, LDH ratio, and risk score emerged as independent prognostic indicators. Table 1 summarized the comprehensive data from the univariate and multivariate analyses. Based on the above data, a nomogram was established to predict the survival probability at 1‐, 3‐, and 5‐year, as is seen in Figure 4A. The time‐dependent ROC curves for this nomogram in the training and validation cohorts are presented in Figure 4B–D. The AUC values for the training cohort at the first, third, and fifth year were 0.812 (95% CI: 0.756–0.868), 0.838 (95% CI: 0.790–0.887), and 0.828 (95% CI: 0.768–0.887), respectively. For the GSE11318 cohort, the AUC values at the first, third, and fifth year were 0.778 (95% CI: 0.685–0.872), 0.809 (95% CI: 0.736–0.882), and 0.818 (95% CI: 0.742–0.894), respectively. In the GSE53786 cohort, the AUC values were 0.840 (95% CI: 0.756–0.925), 0.872 (95% CI: 0.781–0.964), and 0.856 (95% CI: 0.732–0.981), respectively. The findings above reveal that the nomogram performs favorably in predicting OS, and should provide a meaningful cognition for the individualized management of DLBCL.

**TABLE 1:** Univariate and multivariate analyses of an 18‐gene‐risk scoring model, clinical characteristics with overall survival.

Variable	Univariate Cox		Multivariate Cox	
	HR (95% CI)	*p*	HR (95% CI)	*p*
Age	1.030 (1.018–1.042)	< 0.001	1.028 (1.013–1.043)	< 0.001
Sex	1.029 (0.756–1.400)	0.856	—	—
ECOG performance score	1.841 (1.568–2.160)	< 0.001	1.481 (1.228–1.787)	< 0.001
Stage	1.509 (1.294–1.760)	< 0.001	1.291 (1.071–1.555)	0.007
Extra nodal sites number	1.219 (1.013–1.467)	0.036	1.024 (0.807–1.300)	0.844
LDH ratio	1.138 (1.095–1.182)	< 0.001	1.098 (1.040–1.160)	< 0.001
Risk score	12.59 (7.917–20.024)	< 0.001	9.280 (5.276–16.325)	< 0.001

*Note:* The conclusions of univariate and multivariate Cox regression demonstrated that the risk score, age, ECOG performance score, stage, and LDH ratio were independent, significant prognostic factors in DLBCL, with the risk score showing greater predictive capacity.

Abbreviations: ECOG, Eastern Cooperative Oncology Group; LDH, lactate dehydrogenase.

### 3.5 GSEA Analysis Between Low‐ and High‐Risk Group

**FIGURE 5:**
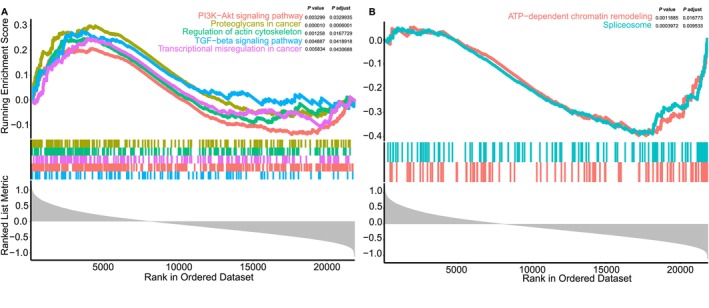
Gene set enrichment analysis. (A) GSEA showing downregulated KEGG gene sets in the low‐risk group, including biosynthesis of cofactors and Epstein–Barr virus infection. (B) GSEA showing upregulated KEGG gene sets in the low‐risk group, including proteoglycans in cancer, oxidative phosphorylation, apelin signaling pathway, chemical carcinogenesis‐reactive oxygen species and the PI3K‐Akt signaling pathway.

DEG analysis and GSEA were conducted among subgroups. Figure 5A,B demonstrated that pathways involving proteoglycans in cancer, PI3K‐Akt signaling pathway, regulation of actin cytoskeleton, TGF‐beta signaling pathway and transcriptional mis‐regulation in cancer had greater enrichment in the low‐risk group. In contrast, pathways such as ATP‐dependent chromatin remodeling and spliceosome were predominantly enriched in the high‐risk group. The result of DEG was illustrated in Figure S3.

### 3.6 Immunological Landscape and ICI‐Related molecules' Differences Between Low‐ and High‐Risk Group

**FIGURE 6:**
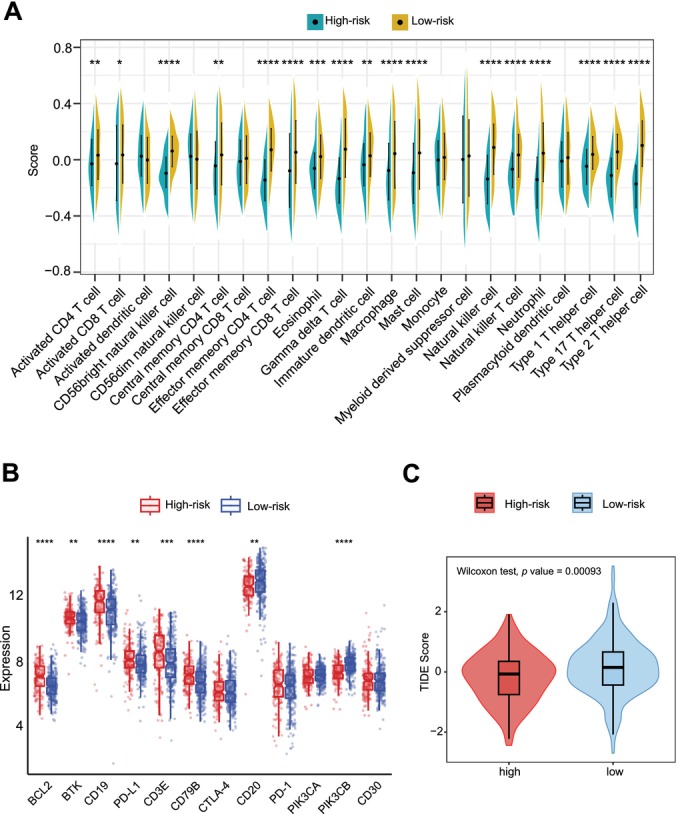
Immune microenvironment analysis and evaluation of response to immunotherapy in both groups. (A) Violin plot displaying differences in immune cell infiltration between the low‐ and high‐risk groups using ssGSEA (****p value < 0.0001,***p value < 0.001, **p value < 0.01; *p value < 0.05). (B) Box plot displaying differences in the levels of expression in targeted‐therapy related genes (PD‐L1, PD‐1, CD3E, CTLA‐4, BCL2, CD30, CD79B, CD19, PIK3CA, PIK3CB and BTK) between the low‐ and high‐risk groups (****p value < 0.0001, ***p value < 0.001, **p value < 0.01; *p value < 0.05). (C) Different TIDE score calculated by TIDE algorithm between two groups.

The differential abundance of infiltrating immune cell are illustrated in Figure 6A. Low‐risk group patients had more enrichment of activated CD4^+^ T cell, activated CD8^+^ T cell, CD56^bright^ nature killer cell(NK cell), central memory CD4^+^ T cell, effector memory CD4^+^ T cell, effector memory CD8^+^ T cell, γδT cell, immature dendritic cell, macrophage, NK cell, NK T cell, Type 1 T helper cell (Th1), Type 2 T helper cell (Th2), Type 17 T helper cell (Th17), eosinophil, mast cell and neutrophil. Since macrophage has been reported that it could act multiple characters in many types of cancers, we also analyzed the components of macrophages. Figure S4A shows a significantly higher level of M2 macrophages, and a lower level of M0 macrophages in the high‐risk group, whereas no notable discrepancy was observed in M1 macrophages. To see how the risk score interacts with the quantities of immune infiltration cells mentioned above, Spearman correlation analyses were performed. Figure S4B exhibits an overview of the correlation analysis. Notably, activated CD4^+^ T cell, CD56^bright^ NK cell, effector memory CD4^+^ T cell, effector memory CD8^+^ T cell, γδT cell, immature dendritic cell, macrophage, NK cell, NK T cell, Th1 cell, Th2 cell, Th17 cell, eosinophil, mast cell and neutrophil consistently showed statistical significance in both differential and correlation analyses, suggesting a strong connection across the risk score and the quantities of immune infiltration cells in DLBCL, especially a negative correlation with the majority of anti‐tumor cells. Figure S5 demonstrates more detailed linear correlations between the abundance of these cells and the risk score. Additionally, we analyzed the expression of immunotherapy and targeted therapy associated molecules across subsets of patients. As illustrated in Figure 6B, the expression levels of PD‐L1, CD20, CD3E, BCL2, BTK, CD19, CD79B, and PI3Kβ exhibited notable disparities between the low‐ and high‐risk patients, whereas no considerable differences were discerned in CTLA‐4, PI3Kα, and PD‐1. It is noteworthy that the expression levels of PD‐L1, CD3E, BCL2, BTK, CD19, and CD79B were significantly higher, whereas the levels of CD20, PI3Kβ were notably lower in high‐risk patients. These results indicate that the high‐risk patients may receive more benefit from therapies that target PD‐L1, CD3E, BCL2, BTK, CD19 and CD79B, while the benefit from CD20‐, PI3K‐targeted therapy may be less pronounced. Finally, the TIDE algorithm was conducted to estimate the responses to immunotherapy on different groups. Figure 6C demonstrates that the high‐risk group had a considerably lower TIDE score (*p* = 0.00093), which implies that high‐risk patients might be less possible to experience immune escape and might be benefited more from immunotherapy.

### 3.7 Drug Sensitivity Analysis

**FIGURE 7:**
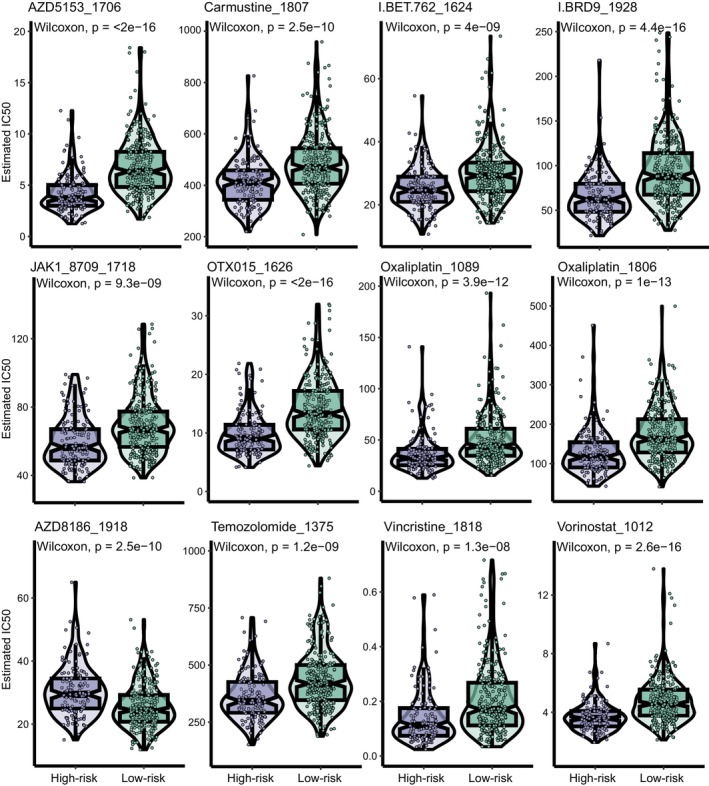
Drug sensitivity analysis between the low‐ and high‐risk groups. Differences in IC50 values of 12 potential drugs (oxaliplatin_1089, oxaliplatin_1806, OTX015_1626, AZD5153_1706, I.BRD9_1928, I.BET.762_1624, JAK1_8709_1718, AZD8186_1918, vincristine_1818, carmustine_1807, temozolomide_1375, and vorinostat_1012) in DLBCL between the low‐ and high‐risk groups.

In this section, we investigate the differential IC50 values of 198 compounds between two groups to provide guidance for drug therapies in different DLBCL patients with the R package “oncoPredict”. Overall, the IC50 values of the 45 compounds differed significantly across groups. These include oxaliplatin_1089, oxaliplatin_1806, OTX015_1626, AZD5153_1706, I.BRD9_1928, I.BET.762_1624, JAK1_8709_1718, AZD8186_1918, vincristine_1818, carmustine_1807, temozolomide_1375, and vorinostat_1012 demonstrates the promising potential for clinical use in DLBCL management, as shown in Figure 7. We also investigated how the risk score correlated with the IC50 values of these selected drugs, as shown in Figure S6C. According to our findings, the high‐risk group's IC50s were lower for oxaliplatin, AZD5153, OTX015, I.BRD9, JAK1_8709, I.BET.762, temozolomide, carmustine and vorinostat, which demonstrated a negative relationship with the risk score. Conversely, AZD8186 had the opposite performance.

### 3.8 Mutational Analysis Between Low‐ and High‐Risk Group

**FIGURE 8:**
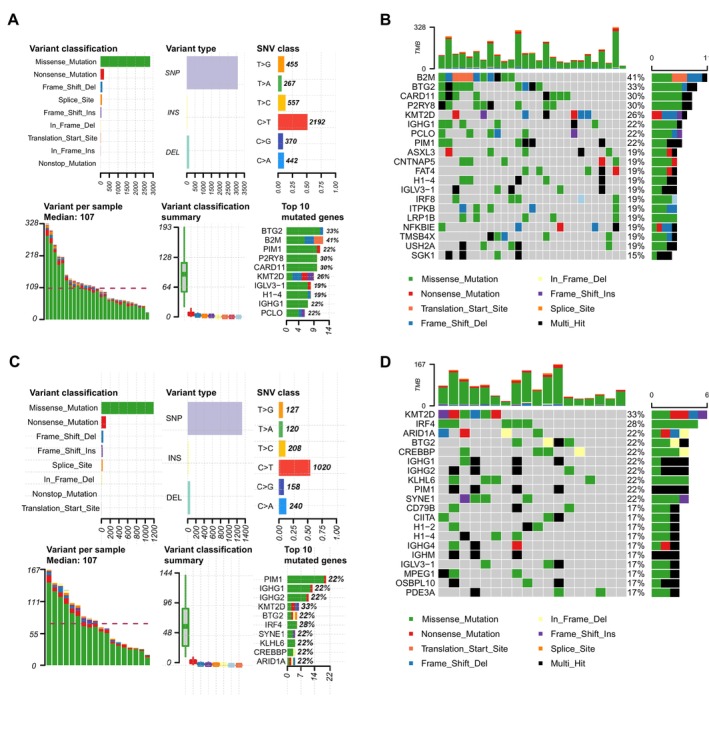
Somatic mutation analysis between the low‐ and high‐risk groups. (A) Somatic mutation profiles of the low‐risk group. (B) Mutation incidence in the low‐risk groups. (C) Somatic mutation profiles of the high‐risk group. (D) Mutation incidence in the high‐risk group.

In this section, we utilized MAF files and transcriptome data (TPM) acquired from TCGA to investigate the differences of somatic mutation across different groups of DLBCL patients. The somatic mutation profiles of the low‐risk group are demonstrated in Figure 8A,B, while the high‐risk group's profiles are demonstrated in Figure 8C,D. Our data suggested that BTG2, KMT2D and PIM1 mutations were prevalent in both groups. However, B2M possessed the most likelihood of mutations in low‐risk patients, while in the other group, KMT2D exhibited the greatest mutation rate. Each patients' tumor mutation burden (TMB) was also calculated and a differential analysis was performed. As shown in Figure S7A,B, no notable discrepancy in TMB was found, however the risk score claimed a significantly negative relationship with TMB.

## 4 Discussion

The mitochondria, essential organelles in living cells, are engaged in a wide range of biological processes. Elucidating the roles mitochondria play in DLBCL may contribute to the development of better therapeutic approaches and aid in identifying which group of patients might benefit from immunotherapy.

Following the application of Lasso algorithm to the training set, 18 genes were identified as prognostic MRGs, including DNM1L, PUSL1, CHCHD4, COX7A1, CPT1A, CYP27A1, POLDIP2, PCK2, MRPL2, PDK3, PDK4, MARC2, ACSM3, COA7, THNSL1, ATAD3B, C15orf48, and TOMM70A. Previous studies showed that dephosphorylation of DNM1L was involved in mitochondrial cleavage and mitochondrial fission. In breast cancer cells, it has been experimentally demonstrated to enhance doxorubicin‐mediated apoptosis by inducing mitochondrial fission [21]. Additionally, in colorectal cancer cells, DNM1L boosts susceptibility to ROS‐induced apoptosis via a akin mechanism, as demonstrated in a previous experiment [22]. CHCHD4, an assembly factor of mitochondrial respiratory chain, has been reported to be associated with histone modification. In multiple cancer cell lines, CHCHD4 depletion has been proven to suppress histone density, thereby altering the susceptibility to histone deacetylase inhibitors [23]. As an important rate‐limiting enzyme in fatty acid oxidation, CPT1A was reported to stabilize c‐Myc and activate the NRF2/GPX4 system to suppress ferroptosis and improve the efficacy of immunotherapy in lung cancer cells [24]. As a cholesterol hydroxylase, CYP27A1 catalyzes the production of 27‐hydroxycholesterol (27OHC), which has been described to impair T‐cell function through its interaction with myeloid cells via liver X receptor [25]. POLDIP2 is a newly identified target of the E7 oncoprotein and studies have shown that it is involved in DNA repairment [26]. PCK2 was found to be downregulated in tumor‐repopulating cells and empowered resistance to ferroptosis in an ACSL4‐dependent manner [27]. The overexpression of COX7A1, a subunit of the cytochrome c oxidase enzyme, was found to render lung cancer cells more susceptible to ferroptosis [28]. Pyruvate dehydrogenase kinases such as PDK3 and PDK4 are important markers in glycolysis, have been reported to be used to predict the recurrence in prostate cancer [29], this might be relevant with the elevated level of lactate [30] and the activation of TNF pathway [31]. Notably, PDK4 has been reported to be involved in drug resistance in DLBCL by activating HDAC8 to reduce CD20 level [32]. Mitochondrial amidoxime reducing Component 2 (MARC2), a newly identified molybdenum enzyme, was found to be downregulated in HCC cells and interacted with p27 to suppress HCC progression [33]. ATAD3B was studied to be a critical role in oxidative stress‐induced mitophagy and clearance of damaged mitochondrial DNA [34]. ACSM3 catalyzes the initiation of lipogenesis by producing acyl‐CoA and has been identified as supporting the growth of prostate cancer cells by protecting against ferroptosis [35]. Reported to be a critical inducer of autophagy, C15orf18 reduces intracellular ATP, activates AMP‐activated protein kinase and upregulates intracellular glutathione levels, thereby reducing oxidative stress and promoting survival [36]. TOMMA70A (also Tom70/TOMM70) was found to be involved in the transport of protein like Gasdermin A3 to maintain mitochondria homeostasis [37]. Study showed that targeting TOMM70A in lung cancer cells could lead to mitochondrial destabilization, then subsequently induce pyroptosis [38].

A categorization of patients as either low‐risk or high‐risk was conducted in accordance with the model. K‐M survival analysis demonstrated a notable difference between two groups, with patients in the high‐risk group exhibiting a shorter OS period. The combination of the ROC curves with the aforementioned data indicated that the model had strong prognostic predictive ability and broad applicability. Moreover, our data suggest that the 18 MRGs risk model exhibited a superior predictive performance compared to other clinical factors as a prognostic signature, followed by the establishment of a nomogram.

Then we performed DEG and GSEA analyses between two groups of patients. The results indicated that the low‐risk group had greater enrichment in the proteoglycans in cancer, PI3K‐Akt signaling pathway, regulation of actin cytoskeleton, TGF‐beta signaling pathway and transcriptional mis‐regulation in cancer, while exhibiting lower enrichment in the ATP‐dependent chromatin remodeling and spliceosome. PI3K‐Akt pathway can be activated by multiple stimulations, such as growth factors and cytokines, which in turn enhances tumor cell survival and their resistance to drugs [39]. Therapies targeting PI3K have established noteworthy therapeutic efficacy [40].

Multiple studies, both experimental and bioinformatic, have sought to elucidate the routines of immune infiltration and the associated gene signatures in DLBCL [41, 42]. However, a clear consensus has yet to be reached. Hence, we accessed the immunological differences among different groups of patients using ssGSEA, CIBERSORT and TIDE algorithm. Our results indicate a tight connection relating the risk score and anti‐tumor effects [43]. Specifically, the abundance scores of activated CD4^+^ T cell, CD56^bright^ NK cell, effector memory CD4^+^ T cell, effector memory CD8^+^ T cell, γδT cell, NK cell, NK T cell, Th1 cell and Th17 cell consistently exhibited negative correlation with the risk score. Moreover, a notable higher level of M2 macrophage was identified in the high‐risk group. CD56^bright^ NK cells, often regarded as “amateur” NK cells in comparison to CD56^dim^ NK cells, have been found to exert a more robust anti‐tumor effect in the presence of IL‐15 [44]. NK cells, NK T cells and γδT cells are non‐MHC‐restricted immune cells which can demonstrate potent cytotoxic activities against cancer cells, and they have gained increasing attention in recent years for their therapeutic potential [45, 46]. CD4^+^, CD8^+^ T cells, as well as Th1 and Th17 cells, are well‐recognized as anti‐tumor immune cells, and their degree of infiltration is positively correlated with prognosis [47, 48, 49]. M2 macrophages have been reported as pro‐tumor factors in many studies, with some suggesting that the heightened recruitment of M2 macrophage contributes to DLBCL progression and resistance to CAR‐T therapy [50, 51].

Furthermore, analyses were performed to ascertain the association between targeted therapy‐relevant molecules and the risk score. Our findings revealed that the high‐risk group exhibited elevated expression levels of PD‐L1, CD3E, BCL2, BTK, CD19, CD79B, and lower expression levels of CD20, PI3Kβ. TIDE analysis also indicated that the high‐risk group might derive greater benefit from immunotherapy. Consequently, the high‐risk patients might hear more from anti‐PD‐L1, anti‐CD3, anti‐BCL2, anti‐CD19, and anti‐CD79B, while potentially benefiting less from anti‐CD20 and anti‐PI3K therapies. The above findings suggest that our model can provide guidance for determining the appropriateness of adding immunotherapy and targeted therapy for individual patients.

The association between IC50 values of drugs and the risk score was explored to offer new insights into treatment selection for different DLBCL patients. Our data suggest that oxaliplatin, vincristine, temozolomide, carmustine, AZD5153, OTX015, I.BRD9, JAK1_8709, and vorinostat might demonstrate better efficacy in treating high‐risk patients. AZD5153, OTX015, I.BET.762, and I.BRD9 belong to bromodomain and extra‐terminal domain (BET) inhibitors, which have showed potent therapeutic effects against DLBCL cells [52]. JAK1_8709 targets JAK1/JAK2, studies have established an excellent efficacy on treating B‐cell lymphoma with anti‐JAK management [53]. As a histone deacetylase inhibitor, vorinostat has been investigated for the treatment of HIV‐associated NHLs [54], one of its counterparts, chidamide, is usually used in the management of relapsed/refractory DLBCL. AZD8186 is a specific PI3Kβ inhibitor, according to our findings above, low‐risk patients might benefit more from PI3Kβ inhibitors [55]. Building on the above findings, launching clinical trials of HDAC and BET inhibitors in the high‐risk patients, as well as PI3K inhibitors in the low‐risk patients, appears both reasonable and promising.

Next, we analyzed the differences in somatic mutation profiles across groups. BTG2 (also known as BTG anti‐proliferation factor), KMT2D (lysine methyltransferase 2D) and PIM1 (serine/threonine‐protein kinase Pim‐1) were commonly mutated in both groups. BTG2 was reported to be involved in the Richter transformation in chronic lymphocytic leukemia [56] and was considered to be one of the molecular labels to classify DLBCL. KMT2D was identified to be one of the most prevalent genetic alterations in DLBCL that raise lymphomagenesis [57]. PIM1 is a special Ser/Thr protein kinase [58], which showed a high mutational frequency in the high‐risk group.

It is pertinent to acknowledge that this study has some constraints. Our analyses relied solely on retrospective data from publicly available sources, without incorporating prospective data from larger cohorts. Existing research highlights regional variations in DLBCL mortality due to the differences in ethnic groups, geographical locations, lifestyles, and other susceptibility factors. Achieving more accurate results will require the collection of detailed clinical information. Meanwhile, more investigations are required to elucidate the molecular pathways through which MRGs influence immunity and survival in DLBCL. Ultimately, the efficacy of the model must be validated in larger populations to ensure its reliability and applicability.

To sum up, this research highlights the importance of MRGs in the progression of DLBCL and introduces an 18 MRGs prognostic model for personalized assessment. Moving forward, this approach could optimize the accuracy of DLBCL prognosis predictions and improve the personalized selection of immunotherapy regimens.

## Author Contributions

**Zhen‐Zhong Zhou:** formal analysis (equal), resources (equal), writing – original draft (equal). **Jia‐Chen Lu:** formal analysis (equal), methodology (equal), writing – original draft (equal). **Song‐Bin Guo:** software (equal), visualization (equal), writing – review and editing (equal). **Xiao‐Peng Tian:** conceptualization (equal), funding acquisition (lead), project administration (lead). **Hai‐Long Li:** data curation (equal), resources (equal), validation (equal). **Hui Zhou:** conceptualization (equal), supervision (equal), supervision (equal). **Wei‐Juan Huang:** methodology (equal), supervision (equal), visualization (equal).

## Conflicts of Interest

The authors declare no conflicts of interest.

## Supporting information


Data S1.

Figure S1.

Figure S2.

Figure S3.

Figure S4.

Figure S5.

Figure S6.

Figure S7.

## Data Availability

The data and materials in this study can be obtained from the corresponding author upon a reasonable request.

## References

[1] S. Susanibar‐Adaniya and S. K. Barta , “2021 Update on Diffuse Large B Cell Lymphoma: A Review of Current Data and Potential Applications on Risk Stratification and Management,” American Journal of Hematology 96 (2021): 617–629. 33661537 10.1002/ajh.26151 PMC8172085

[2] A. P. Dabrowska‐Iwanicka and G. S. Nowakowski , “DLBCL: Who Is High Risk and How Should Treatment Be Optimized?,” Blood 144, no. 25 (2023): 2573–2582. 10.1182/blood.2023020779 37922443

[3] X.‐X. Zheng , J.‐J. Chen , Y.‐B. Sun , T. Q. Chen , J. Wang , and S. C. Yu , “Mitochondria in Cancer Stem Cells: Achilles Heel or Hard Armor,” Trends in Cell Biology 33 (2023): 708–727. 37137792 10.1016/j.tcb.2023.03.009

[4] H. Zhang , X. Yu , J. Ye , et al., “Systematic Investigation of Mitochondrial Transfer Between Cancer Cells and T Cells at Single‐Cell Resolution,” Cancer Cell 41 (2023): 1788–1802.e10. 37816332 10.1016/j.ccell.2023.09.003 PMC10568073

[5] J. M. Winter , T. Yadav , and J. Rutter , “Stressed to Death: Mitochondrial Stress Responses Connect Respiration and Apoptosis in Cancer,” Molecular Cell 82 (2022): 3321–3332. 35961309 10.1016/j.molcel.2022.07.012 PMC9481690

[6] H. Jiang , H. Fu , Y. Guo , P. Hu , and J. Shi , “Evoking Tumor Associated Macrophages by Mitochondria‐Targeted Magnetothermal Immunogenic Cell Death for Cancer Immunotherapy,” Biomaterials 289 (2022): 121799. 36152515 10.1016/j.biomaterials.2022.121799

[7] D. C. Watson , D. Bayik , S. Storevik , et al., “GAP43‐Dependent Mitochondria Transfer From Astrocytes Enhances Glioblastoma Tumorigenicity,” Nature Cancer 4 (2023): 648–664. 37169842 10.1038/s43018-023-00556-5 PMC10212766

[8] S. B. Guo , S. Du , K. Y. Cai , H. J. Cai , W. J. Huang , and X. P. Tian , “A Scientometrics and Visualization Analysis of Oxidative Stress Modulator Nrf2 in Cancer Profiles Its Characteristics and Reveals Its Association With Immune Response,” Heliyon 9 (2023): e17075. 37342570 10.1016/j.heliyon.2023.e17075 PMC10277599

[9] P. Wei , A. J. Bott , A. A. Cluntun , et al., “Mitochondrial Pyruvate Supports Lymphoma Proliferation by Fueling a Glutamate Pyruvate Transaminase 2‐Dependent Glutaminolysis Pathway,” Science Advances 8 (2022): eabq0117. 36179030 10.1126/sciadv.abq0117 PMC9524954

[10] W. T. Wang , T. Y. Xing , K. X. Du , et al., “CD30 Protects EBV‐Positive Diffuse Large B‐Cell Lymphoma Cells Against Mitochondrial Dysfunction Through BNIP3‐Mediated Mitophagy,” Cancer Letters 583 (2024): 216616. 38211650 10.1016/j.canlet.2024.216616

[11] M. Li , Y.‐L. Chiang , C. A. Lyssiotis , et al., “Non‐Oncogene Addiction to SIRT3 Plays a Critical Role in Lymphomagenesis,” Cancer Cell 35 (2019): 916–931.e9. 31185214 10.1016/j.ccell.2019.05.002 PMC7534582

[12] M. Li , M. R. Teater , J. Y. Hong , et al., “Translational Activation of ATF4 Through Mitochondrial Anaplerotic Metabolic Pathways Is Required for DLBCL Growth and Survival,” Blood Cancer Discovery 3 (2022): 50–65. 35019856 10.1158/2643-3230.BCD-20-0183 PMC9789686

[13] L. C. Cerchietti , S. N. Yang , R. Shaknovich , et al., “A Peptomimetic Inhibitor of BCL6 With Potent Antilymphoma Effects In Vitro and In Vivo,” Blood 113 (2009): 3397–3405. 18927431 10.1182/blood-2008-07-168773 PMC2668844

[14] P. Caro , A. U. Kishan , E. Norberg , et al., “Metabolic Signatures Uncover Distinct Targets in Molecular Subsets of Diffuse Large B Cell Lymphoma,” Cancer Cell 22 (2012): 547–560. 23079663 10.1016/j.ccr.2012.08.014 PMC3479446

[15] Y. Liu , S. Kimpara , N. M. Hoang , et al., “EGR1‐Mediated Metabolic Reprogramming to Oxidative Phosphorylation Contributes to Ibrutinib Resistance in B‐Cell Lymphoma,” Blood 142 (2023): 1879–1894. 37738652 10.1182/blood.2023020142 PMC10731920

[16] A. Tedeschi , A. M. Frustaci , A. Condoluci , et al., “Atezolizumab, Venetoclax, and Obinutuzumab Combination in Richter Transformation Diffuse Large B‐Cell Lymphoma (MOLTO): A Multicentre, Single‐Arm, Phase 2 Trial,” Lancet Oncology 25 (2024): 1298–1309. 39270702 10.1016/S1470-2045(24)00396-6

[17] K. Manos , G. Chong , C. Keane , et al., “Immune Priming With Avelumab and Rituximab Prior to R‐CHOP in Diffuse Large B‐Cell Lymphoma: The Phase II AvR‐CHOP Study,” Leukemia 37 (2023): 1092–1102. 36906715 10.1038/s41375-023-01863-7

[18] S.‐B. Guo , L.‐S. Hu , W.‐J. Huang , Z. Z. Zhou , H. Y. Luo , and X. P. Tian , “Comparative Investigation of Neoadjuvant Immunotherapy Versus Adjuvant Immunotherapy in Perioperative Patients With Cancer: A Global‐Scale, Cross‐Sectional, and Large‐Sample Informatics Study,” International Journal of Surgery 110 (2024): 4660–4671. 38652128 10.1097/JS9.0000000000001479 PMC11325894

[19] S. Rath , R. Sharma , R. Gupta , et al., “MitoCarta3.0: An Updated Mitochondrial Proteome Now With Sub‐Organelle Localization and Pathway Annotations,” Nucleic Acids Research 49 (2020): D1541–D1547. 10.1093/nar/gkaa1011 PMC7778944 33174596

[20] B. Ru , C. N. Wong , Y. Tong , et al., “TISIDB: An Integrated Repository Portal for Tumor–Immune System Interactions,” Bioinformatics 35 (2019): 4200–4202. 30903160 10.1093/bioinformatics/btz210

[21] J. Zhou , G. Li , Y. Zheng , et al., “A Novel Autophagy/Mitophagy Inhibitor Liensinine Sensitizes Breast Cancer Cells to Chemotherapy Through DNM1L‐Mediated Mitochondrial Fission,” Autophagy 11 (2015): 1259–1279. 26114658 10.1080/15548627.2015.1056970 PMC4590597

[22] K. Iskandar , J. Foo , A. Q. X. Liew , et al., “A Novel MTORC2‐AKT‐ROS Axis Triggers Mitofission and Mitophagy‐Associated Execution of Colorectal Cancer Cells Upon Drug‐Induced Activation of Mutant KRAS,” Autophagy 20 (2024): 1418–1441. 38261660 10.1080/15548627.2024.2307224 PMC11210925

[23] C. Bruhn , G. Bastianello , and M. Foiani , “Cancer Cell Histone Density Links Global Histone Acetylation, Mitochondrial Proteome and Histone Acetylase Inhibitor Sensitivity,” Communications Biology 5 (2022): 882. 36030322 10.1038/s42003-022-03846-3 PMC9420116

[24] L. Ma , C. Chen , C. Zhao , et al., “Targeting Carnitine Palmitoyl Transferase 1A (CPT1A) Induces Ferroptosis and Synergizes With Immunotherapy in Lung Cancer,” Signal Transduction and Targeted Therapy 9 (2024): 64. 38453925 10.1038/s41392-024-01772-w PMC10920667

[25] L. Ma , L. Wang , A. T. Nelson , et al., “27‐Hydroxycholesterol Acts on Myeloid Immune Cells to Induce T Cell Dysfunction, Promoting Breast Cancer Progression,” Cancer Letters 493 (2020): 266–283. 32861706 10.1016/j.canlet.2020.08.020 PMC7572761

[26] D. Bruyere , P. Roncarati , A. Lebeau , et al., “Human Papillomavirus E6/E7 Oncoproteins Promote Radiotherapy‐Mediated Tumor Suppression by Globally Hijacking Host DNA Damage Repair,” Theranostics 13 (2023): 1130–1149. 36793865 10.7150/thno.78091 PMC9925306

[27] Z. Li , Z.‐M. Xu , W.‐P. Chen , et al., “Tumor‐Repopulating Cells Evade Ferroptosis via PCK2‐Dependent Phospholipid Remodeling,” Nature Chemical Biology 20 (2024): 1341–1352. 38720107 10.1038/s41589-024-01612-6 PMC11427348

[28] Y. Feng , J. Xu , M. Shi , et al., “COX7A1 Enhances the Sensitivity of Human NSCLC Cells to Cystine Deprivation‐Induced Ferroptosis via Regulating Mitochondrial Metabolism,” Cell Death & Disease 13 (2022): 988. 36418320 10.1038/s41419-022-05430-3 PMC9684511

[29] M. Oberhuber , M. Pecoraro , M. Rusz , et al., “STAT3‐Dependent Analysis Reveals PDK4 as Independent Predictor of Recurrence in Prostate Cancer,” Molecular Systems Biology 16 (2020): e9247. 32323921 10.15252/msb.20199247 PMC7178451

[30] X. Dou , Q. Fu , Q. Long , et al., “PDK4‐Dependent Hypercatabolism and Lactate Production of Senescent Cells Promotes Cancer Malignancy,” Nature Metabolism 5 (2023): 1887–1910. 10.1038/s42255-023-00912-w PMC10663165 37903887

[31] N. E. Boutagy , J. W. Fowler , K. A. Grabinska , et al., “TNFα Increases the Degradation of Pyruvate Dehydrogenase Kinase 4 by the Lon Protease to Support Proinflammatory Genes,” Proceedings of the National Academy of Sciences of the United States of America 120 (2023): e2218150120. 37695914 10.1073/pnas.2218150120 PMC10515159

[32] X. Wu , C. Ban , W. Deng , et al., “Unveiling the PDK4‐Centered Rituximab‐Resistant Mechanism in DLBCL: The Potential of the ‘Smart’ Exosome Nanoparticle Therapy,” Molecular Cancer 23 (2024): 144. 39004737 10.1186/s12943-024-02057-0 PMC11247735

[33] D. Wu , Y. Wang , G. Yang , et al., “A Novel Mitochondrial Amidoxime Reducing Component 2 Is a Favorable Indicator of Cancer and Suppresses the Progression of Hepatocellular Carcinoma by Regulating the Expression of P27,” Oncogene 39 (2020): 6099–6112. 32811980 10.1038/s41388-020-01417-6 PMC7498369

[34] L. Shu , C. Hu , M. Xu , et al., “ATAD3B Is a Mitophagy Receptor Mediating Clearance of Oxidative Stress‐Induced Damaged Mitochondrial DNA,” EMBO Journal 40 (2021): e106283. 33665835 10.15252/embj.2020106283 PMC8047441

[35] R. K. Shrestha , Z. D. Nassar , A. R. Hanson , et al., “ACSM1 and ACSM3 Regulate Fatty Acid Metabolism to Support Prostate Cancer Growth and Constrain Ferroptosis,” Cancer Research 84 (2024): 2313–2332. 38657108 10.1158/0008-5472.CAN-23-1489

[36] Y. Takakura , M. Machida , N. Terada , et al., “Mitochondrial Protein C15ORF48 Is a Stress‐Independent Inducer of Autophagy That Regulates Oxidative Stress and Autoimmunity,” Nature Communications 15 (2024): 953. 10.1038/s41467-024-45206-1 PMC10831050 38296961

[37] C. F. Bennett , P. Latorre‐Muro , and P. Puigserver , “Mechanisms of Mitochondrial Respiratory Adaptation,” Nature Reviews Molecular Cell Biology 23 (2022): 817–835. 35804199 10.1038/s41580-022-00506-6 PMC9926497

[38] L.‐G. Li , J. Hu , N. Han , et al., “Dihydroartemisinin‐Driven TOM70 Inhibition Leads to Mitochondrial Destabilization to Induce Pyroptosis Against Lung Cancer,” Phytotherapy Research 38 (2024): 3856–3876. 38761036 10.1002/ptr.8242

[39] M. Pi , H. Kuang , C. Yue , et al., “Targeting Metabolism to Overcome Cancer Drug Resistance: A Promising Therapeutic Strategy for Diffuse Large B Cell Lymphoma,” Drug Resistance Updates 61 (2022): 100822. 35257981 10.1016/j.drup.2022.100822

[40] W. Xu , P. Berning , and G. Lenz , “Targeting B‐Cell Receptor and PI3K Signaling in Diffuse Large B‐Cell Lymphoma,” Blood 138 (2021): 1110–1119. 34320160 10.1182/blood.2020006784

[41] Z. Y. Xu‐Monette , L. Wei , X. Fang , et al., “Genetic Subtyping and Phenotypic Characterization of the Immune Microenvironment and MYC/BCL2 Double Expression Reveal Heterogeneity in Diffuse Large B‐Cell Lymphoma,” Clinical Cancer Research 28 (2022): 972–983. 34980601 10.1158/1078-0432.CCR-21-2949 PMC9137388

[42] C. Zhang , Q. Lin , C. Li , Y. Qiu , J. Y. Chen , and X. P. Zhu , “Comprehensive Analysis of the Prognostic Implication and Immune Infiltration of CISD2 in Diffuse Large B‐Cell Lymphoma,” Frontiers in Immunology 14 (2023): 1277695. 38155967 10.3389/fimmu.2023.1277695 PMC10754510

[43] Q. Jia , W. Wu , Y. Wang , et al., “Local Mutational Diversity Drives Intratumoral Immune Heterogeneity in Non‐Small Cell Lung Cancer,” Nature Communications 9 (2018): 5361. 10.1038/s41467-018-07767-w PMC6299138 30560866

[44] J. A. Wagner , M. Rosario , R. Romee , et al., “CD56bright NK Cells Exhibit Potent Antitumor Responses Following IL‐15 Priming,” Journal of Clinical Investigation 127 (2017): 4042–4058. 28972539 10.1172/JCI90387 PMC5663359

[45] N. Shimasaki , A. Jain , and D. Campana , “NK Cells for Cancer Immunotherapy,” Nature Reviews Drug Discovery 19 (2020): 200–218. 31907401 10.1038/s41573-019-0052-1

[46] A. Fenis , O. Demaria , L. Gauthier , E. Vivier , and E. Narni‐Mancinelli , “New Immune Cell Engagers for Cancer Immunotherapy,” Nature Reviews Immunology 24 (2024): 471–486. 10.1038/s41577-023-00982-7 38273127

[47] D. E. Speiser , O. Chijioke , K. Schaeuble , and C. Münz , “CD4+ T Cells in Cancer,” Nature Cancer 4 (2023): 317–329. 36894637 10.1038/s43018-023-00521-2

[48] J. R. Giles , A.‐M. Globig , S. M. Kaech , and E. J. Wherry , “CD8+ T Cells in the Cancer‐Immunity Cycle,” Immunity 56 (2023): 2231–2253. 37820583 10.1016/j.immuni.2023.09.005 PMC11237652

[49] J. Lee , B. Lozano‐Ruiz , F. M. Yang , D. D. Fan , L. Shen , and J. M. González‐Navajas , “The Multifaceted Role of Th1, Th9, and Th17 Cells in Immune Checkpoint Inhibition Therapy,” Frontiers in Immunology 12 (2021): 625667. 33777008 10.3389/fimmu.2021.625667 PMC7994325

[50] Y.‐H. Huang , K. Cai , P.‐P. Xu , et al., “CREBBP/EP300 Mutations Promoted Tumor Progression in Diffuse Large B‐Cell Lymphoma Through Altering Tumor‐Associated Macrophage Polarization via FBXW7‐NOTCH‐CCL2/CSF1 Axis,” Signal Transduction and Targeted Therapy 6 (2021): 10. 33431788 10.1038/s41392-020-00437-8 PMC7801454

[51] Z.‐X. Yan , Y. Dong , N. Qiao , et al., “Cholesterol Efflux From C1QB‐Expressing Macrophages Is Associated With Resistance to Chimeric Antigen Receptor T Cell Therapy in Primary Refractory Diffuse Large B Cell Lymphoma,” Nature Communications 15 (2024): 5183. 10.1038/s41467-024-49495-4 PMC11189439 38890370

[52] A. Schmitt , M. Grimm , N. Kreienkamp , et al., “BRD4 Inhibition Sensitizes Diffuse Large B‐Cell Lymphoma Cells to Ferroptosis,” Blood 142 (2023): 1143–1155. 37294920 10.1182/blood.2022019274

[53] J. Zak , I. Pratumchai , B. S. Marro , et al., “JAK Inhibition Enhances Checkpoint Blockade Immunotherapy in Patients With Hodgkin Lymphoma,” Science 384 (2024): eade8520. 38900864 10.1126/science.ade8520 PMC11283877

[54] J. C. Ramos , J. A. Sparano , A. Chadburn , et al., “Impact of Myc in HIV‐Associated Non‐Hodgkin Lymphomas Treated With EPOCH and Outcomes With Vorinostat (AMC‐075 Trial),” Blood 136 (2020): 1284–1297. 32430507 10.1182/blood.2019003959 PMC7483436

[55] W. Xu , P. Berning , T. Erdmann , et al., “mTOR Inhibition Amplifies the Anti‐Lymphoma Effect of PI3Kβ/δ Blockage in Diffuse Large B‐Cell Lymphoma,” Leukemia 37 (2023): 178–189. 36352190 10.1038/s41375-022-01749-0 PMC9883168

[56] J. Klintman , N. Appleby , B. Stamatopoulos , et al., “Genomic and Transcriptomic Correlates of Richter Transformation in Chronic Lymphocytic Leukemia,” Blood 137 (2021): 2800–2816. 33206936 10.1182/blood.2020005650 PMC8163497

[57] M. Reimann , J. Schrezenmeier , P. Richter‐Pechanska , et al., “Adaptive T‐Cell Immunity Controls Senescence‐Prone MyD88‐ or CARD11‐Mutant B‐Cell Lymphomas,” Blood 137 (2021): 2785–2799. 33232972 10.1182/blood.2020005244

[58] X. Yi , Z. Cao , Y. Yuan , et al., “Design and Synthesis of a Novel Mitochondria‐Targeted Osteosarcoma Theranostic Agent Based on a PIM1 Kinase Inhibitor,” Journal of Controlled Release 332 (2021): 434–447. 33662457 10.1016/j.jconrel.2021.02.028

