# Supplementary figures and images for: A Mitochondria‐Related Signature in Diffuse Large B‐Cell Lymphoma: Prognosis, Immune and Therapeutic Features
Source: Cancer Med. 2025 Jan 15;14(2):e70602. doi: 10.1002/cam4.70602 (PMC11733595; doi:10.1002/cam4.70602)

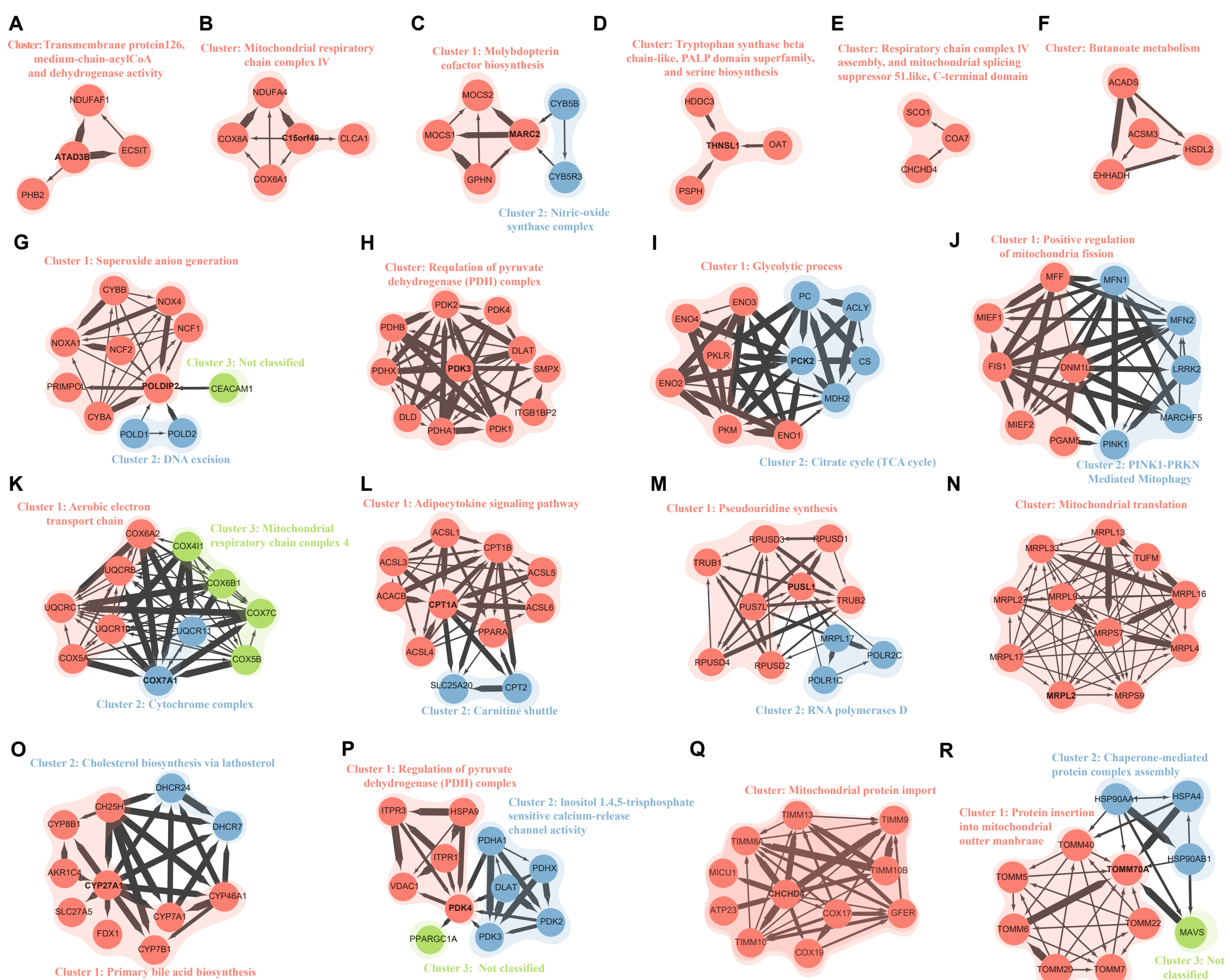

Supplementary Figure 1

Supplement: Supplementary file 2 — Figure S1. [file CAM4-14-e70602-s006.pdf]

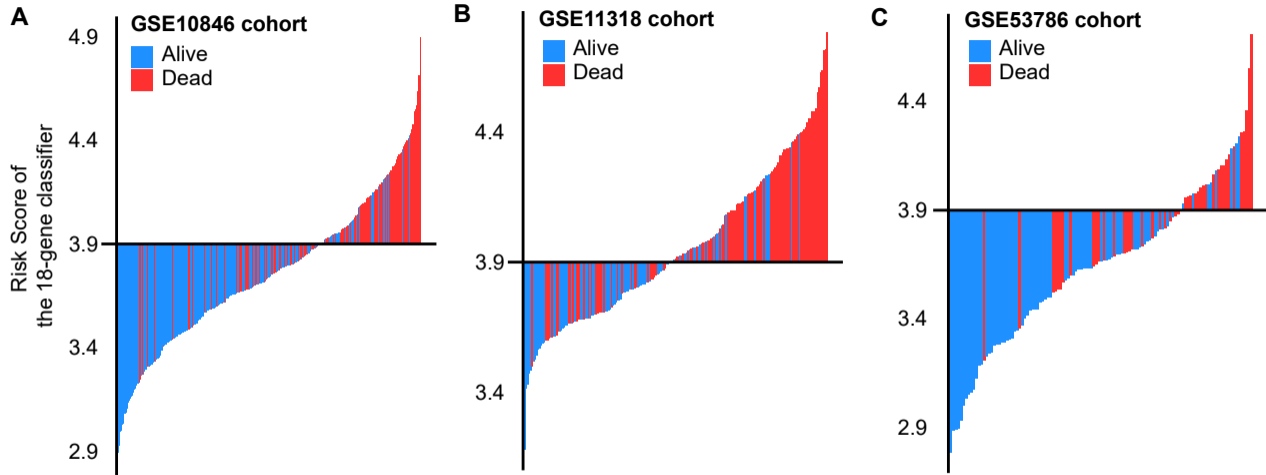

Supplementary Figure 2

Supplement: Supplementary file 3 — Figure S2. [file CAM4-14-e70602-s007.pdf]

● NS ● Log<sub>2</sub> FC ● P value ● P value and log<sub>2</sub> FC

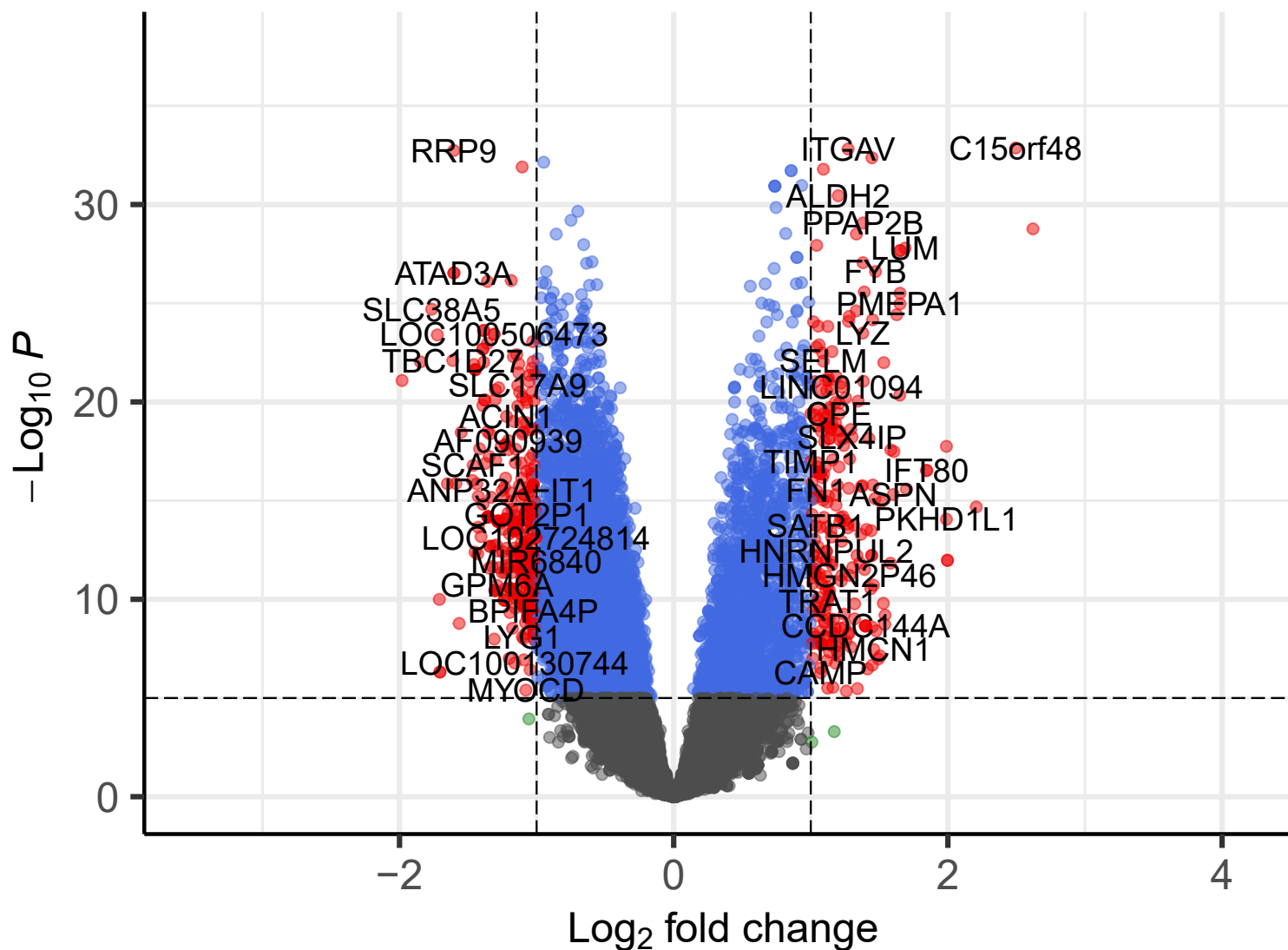

**Supplementary Figure 3**

Supplement: Supplementary file 4 — Figure S3. [file CAM4-14-e70602-s004.pdf]

**A**

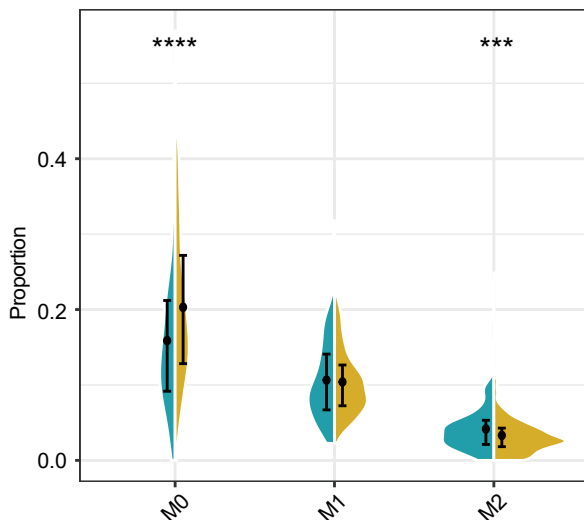

**B**

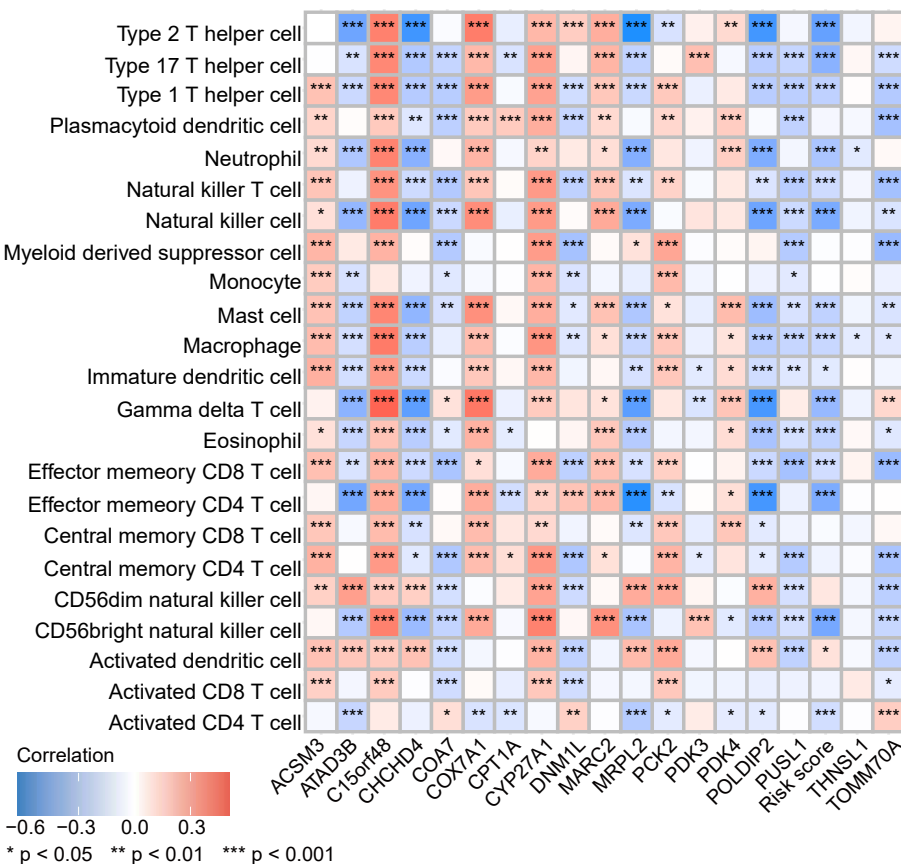

**Supplementary Figure 4**

Supplement: Supplementary file 5 — Figure S4. [file CAM4-14-e70602-s005.pdf]

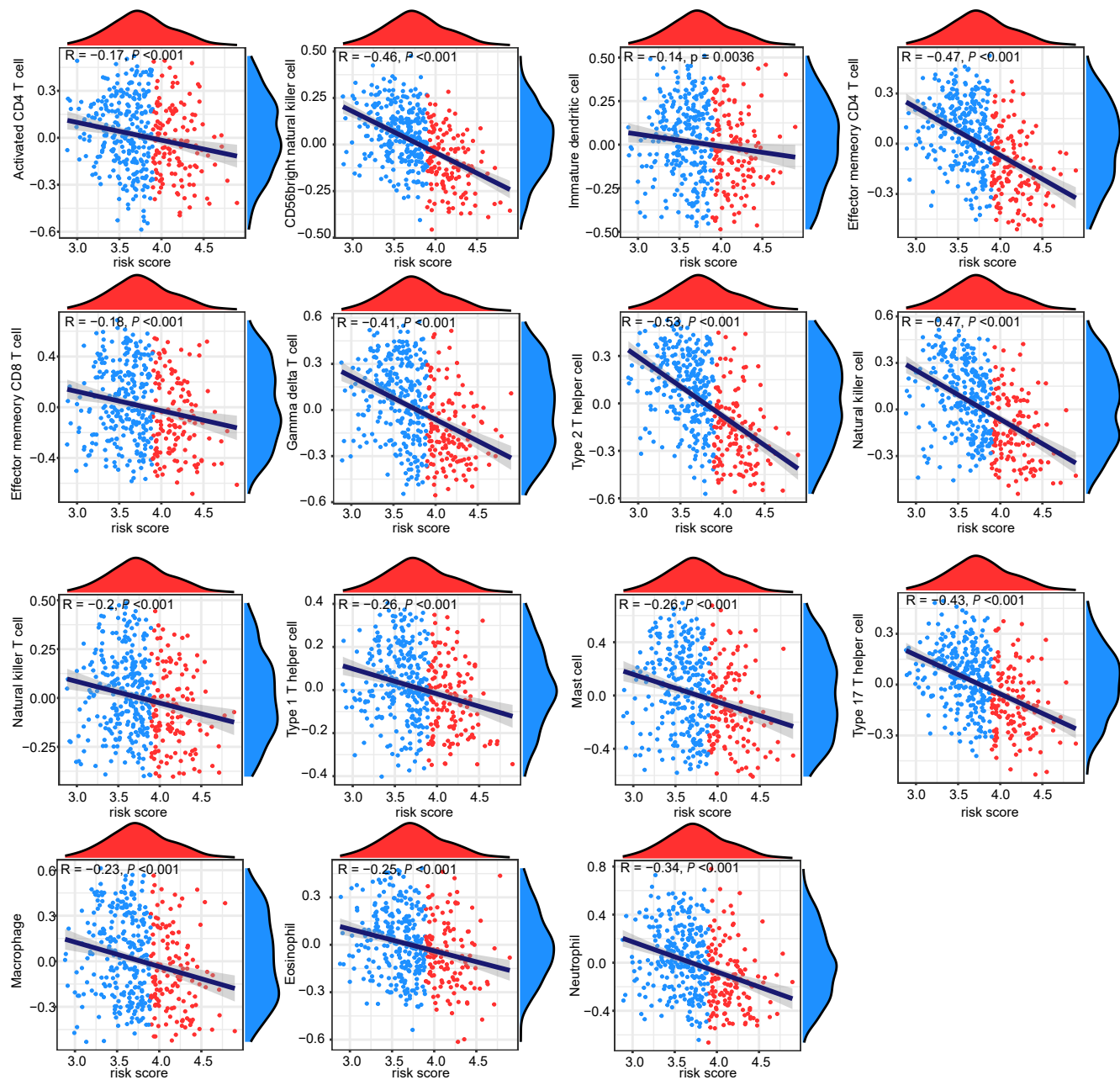

**Supplementary Figure 5**

Supplement: Supplementary file 6 — Figure S5. [file CAM4-14-e70602-s008.pdf]

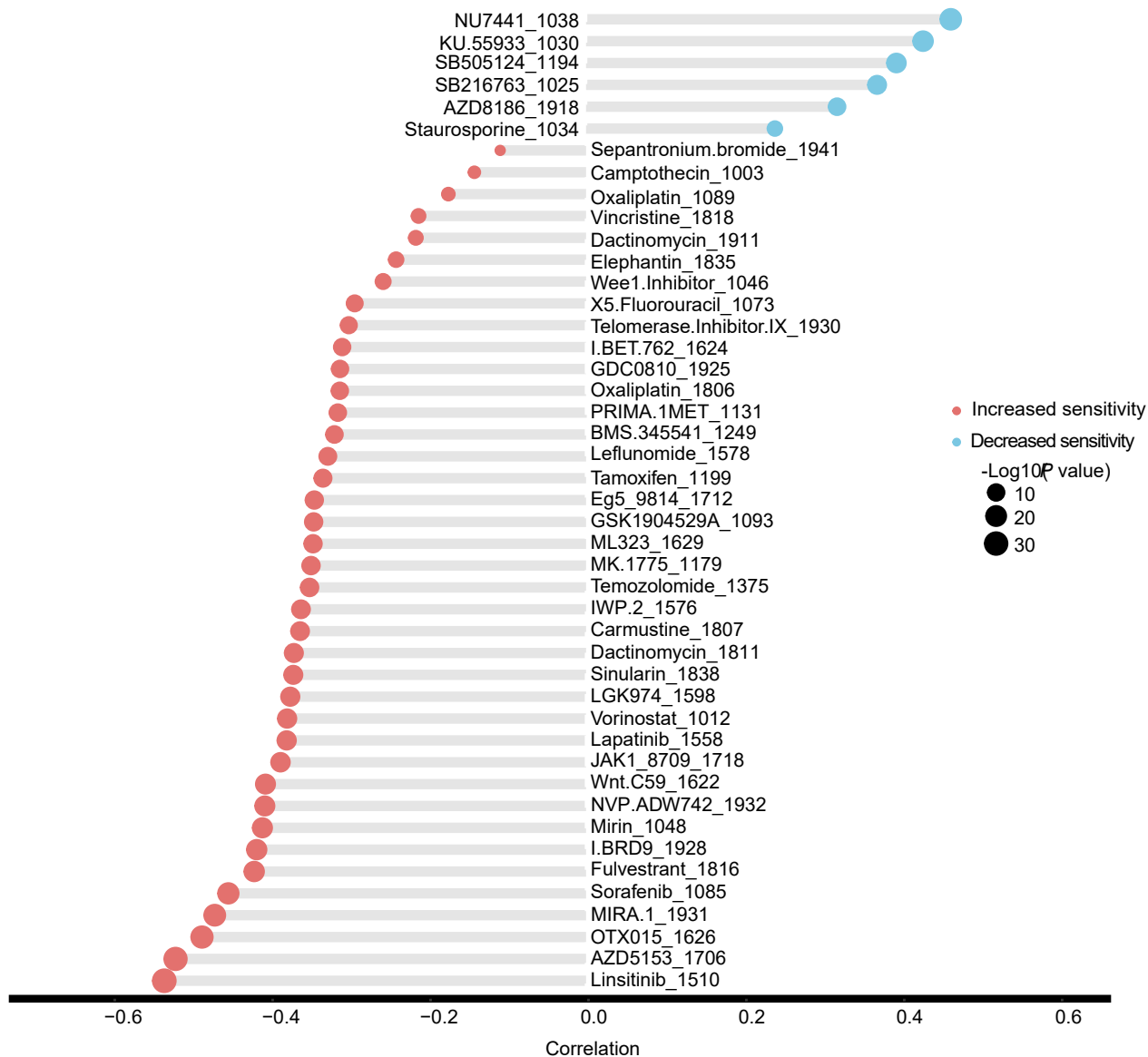

**Supplementary Figure 6**

Supplement: Supplementary file 7 — Figure S6. [file CAM4-14-e70602-s001.pdf]

**A**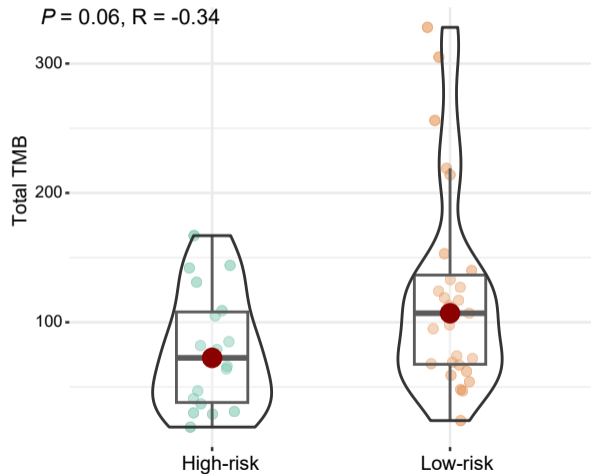**B**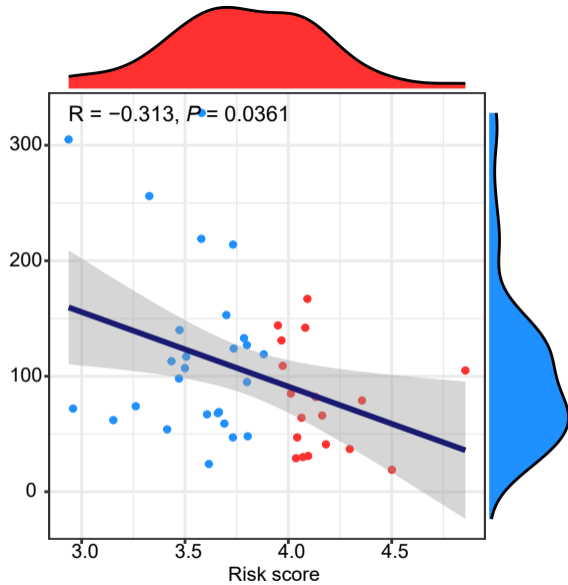**Supplementary Figure 7**

Supplement: Supplementary file 8 — Figure S7. [file CAM4-14-e70602-s002.pdf]
